# Variation in Post-Transplant Cancer Incidence among Italian Kidney Transplant Recipients over a 25-Year Period

**DOI:** 10.3390/cancers15041347

**Published:** 2023-02-20

**Authors:** Pierluca Piselli, Diego Serraino, Claudia Cimaglia, Lucrezia Furian, Luigi Biancone, Ghil Busnach, Nicola Bossini, Paola Todeschini, Maurizio Iaria, Franco Citterio, Mariarosaria Campise, Massimiliano Veroux, Giuseppe Tisone, Vincenzo Cantaluppi, Margherita Mangino, Simona Simone, Davide Argiolas, Andrea Ambrosini, Francesco Pisani, Flavia Caputo, Martina Taborelli

**Affiliations:** 1 Department of Epidemiology and Pre-Clinical Research, National Institute for Infectious Diseases “L. Spallanzani” IRCCS, 00149 Rome, Italy; 2 Unit of Cancer Epidemiology, Centro di Riferimento Oncologico di Aviano (CRO), IRCCS, 33081 Aviano, Italy; 3 Unit of Kidney and Pancreas Transplantation, Department of Surgical, Oncological and Gastroenterological Sciences, University of Padova, 35128 Padova, Italy; 4 Division of Nephrology Dialysis and Transplantation, Renal Transplantation Center “A. Vercellone”, Città della Salute e della Scienza University Hospital, 10126 Turin, Italy; 5 Grande Ospedale Metropolitano Niguarda, 20162 Milano, Italy; 6 Unit of Nephrology, ASST Spedali Civili di Brescia, 25123 Brescia, Italy; 7 Department of Experimental Diagnostic and Specialty Medicine (DIMES), Nephrology, Dialysis and Renal Transplant Unit, IRCCS S. Orsola Hospital, University of Bologna, 40138 Bologna, Italy; 8 Department of General and Specialized Surgery, Division of General Surgery, Transplant Surgery Unit, Parma University Hospital, 43126 Parma, Italy; 9 Department of Surgery, Fondazione Policlinico Universitario A. Gemelli IRCCS, 00168 Rome, Italy; 10 Unit of Nephrology, Dialysis, and Renal Transplantation, Fondazione IRCCS Ca’ Granda Ospedale Maggiore Policlinico, 20122 Milano, Italy; 11 Organ Transplantation Unit, Department of Medical and Surgical Sciences and Advanced Technologies, University Hospital of Catania, 95123 Catania, Italy; 12 UOC Transplant Unit, Department of Surgery, Tor Vergata University, 00133 Rome, Italy; 13 Nephrology and Kidney Transplantation Unit, Department of Translational Medicine, University of Piemonte Orientale (UPO), “Maggiore della Carità” University Hospital, 28100 Novara, Italy; 14 Nephrology, Dialysis, Transplantation Unit, Ca’ Foncello Hospital, 31100 Treviso, Italy; 15 Nephrology, Dialysis and Transplantation Unit, Department of Emergency and Organ Transplantation, University of Bari, 70124 Bari, Italy; 16 Renal Transplant Unit, Azienda Ospedaliera Brotzu, 09047 Cagliari, Italy; 17 Renal Transplant Unit, Azienda Ospedaliera Ospedale di Circolo e Fondazione Macchi, 21100 Varese, Italy; 18 General and Transplant Surgery Department, University of L’Aquila, 67100 L’Aquila, Italy; 19 Nephrology Dialysis and Renal Transplant Department, Civico and Di Cristina Hospital, 90127 Palermo, Italy

**Keywords:** kidney transplant, immunosuppression, virus-related malignancy, trend, Italy, cohort study

## Abstract

**Simple Summary:**

Solid-organ transplant recipients are known to be at higher risk of developing several cancer types, mainly virus-related malignancies. Monitoring trends in the incidence of post-transplant cancers among individuals who received solid organ transplantation helps to improve preventive measures and outcomes. This cohort study aimed to examine, over a 25-year period in Italy, variations in the occurrence of post-transplant cancers among 11,418 recipients of kidney transplantation (KT). Cancer incidence over three periods (1997–2004; 2005–2012; and 2013–2021) was analyzed within the cohort and in comparison with the general population. After multivariate adjustment, both approaches highlighted reduced risks of Kaposi’s sarcoma, whereas no statistically significant changes over time in the incidence of other cancers were noted. Accordingly, the results of this study highlighted the need to sustain and strengthen cancer-preventive actions in KT recipients.

**Abstract:**

This cohort study examined 25-year variations in cancer incidence among 11,418 Italian recipients of kidney transplantation (KT) from 17 Italian centers. Cancer incidence was examined over three periods (1997–2004; 2005–2012; and 2013–2021) by internal (Incidence rate ratio-IRR) and external (standardized incidence ratios-SIR) comparisons. Poisson regression was used to assess trends. Overall, 1646 post-transplant cancers were diagnosed, with incidence rates/1000 person-years ranging from 15.5 in 1997–2004 to 21.0 in 2013–2021. Adjusted IRRs showed a significant reduction in incidence rates across periods for all cancers combined after exclusion of nonmelanoma skin cancers (IRR = 0.90, 95% confidence interval-CI: 0.76–1.07 in 2005–2012; IRR = 0.72, 95% CI: 0.60–0.87 in 2013–2021 vs. 1997–2004; P_trend_ < 0.01). In site-specific analyses, however, significant changes in incidence rates were observed only for Kaposi’s sarcoma (KS; IRR = 0.37, 95% CI: 0.24–0.57 in 2005–2012; IRR = 0.09, 95% CI: 0.04–0.18 in 2013–2021; P_trend_ < 0.01). As compared to the general population, the overall post-transplant cancer risk in KT recipients was elevated, with a decreasing magnitude over time (SIR = 2.54, 95% CI: 2.26–2.85 in 1997–2004; SIR = 1.99, 95% CI: 1.83–2.16 in 2013–2021; P_trend_ < 0.01). A decline in SIRs was observed specifically for non-Hodgkin lymphoma and KS, though only the KS trend retained statistical significance after adjustment. In conclusion, apart from KS, no changes in the incidence of other cancers over time were observed among Italian KT recipients.

## 1. Introduction

The risk of developing cancer is two to five times higher in individuals who received solid organ transplantation (SOT) than in the corresponding general population [1,2,3,4,5]. Furthermore, several studies have indicated that de novo cancers among SOT recipients tend to be clinically more aggressive and to have a poorer prognosis [6,7,8].

Immunosuppressive drugs have long been recognized as a major contributing factor driving the increased risk of post-transplant cancers [9,10]. Indeed, immunosuppression impairs the ability of the host immune system to control oncogenic viruses, leading to an increased risk of developing virus-related tumors including Kaposi’s sarcoma (KS) and non-Hodgkin lymphomas (NHL) [11]. In addition, the risk of some other cancers not associated with viral infections (e.g., melanoma, lung, and head and neck cancers) has been reported to be increased after SOT, likely attributable—in addition to the iatrogenic immune suppression—to behavioral risk factors (e.g., heavy alcohol consumption, smoking) and/or factors related to the underlying disease [12,13].

In view of the growing transplant activity, monitoring trends in cancer incidence among SOT recipients offers the opportunity to update cancer-preventive measures and, thus, to optimize long-term patient outcomes. Variations over time in cancer incidence may reflect factors directly related to transplantation itself (e.g., changes in immunosuppressive treatments or other drug effects), as well as mirror trends in the general population.

Although several large time-trend investigations have been performed in the United States [14] and Northern Europe [1,15], scanty evidence has been published in southern European countries, including Italy. The aim of this study was to examine variations in the incidence of cancers among Italian recipients of kidney transplantation (KT) over a 25-year period.

## 2. Materials and Methods

A cohort study has been conducted among individuals undergoing KT between 1997 and 2021 in 17 centers throughout Italy. The recruitment started in 1997 and it is permanently open (this is a dynamic cohort), i.e., KT recipients can enter the cohort (when they meet eligibility criteria) at different times during the study period.

**Figure 1:**
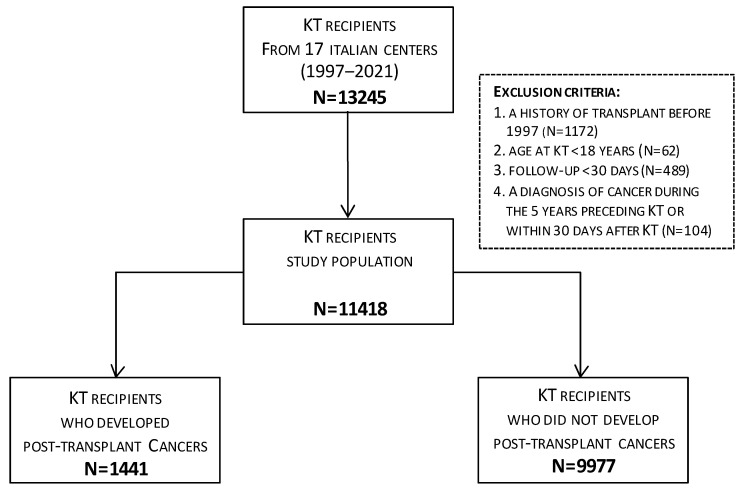
Flow chart of kidney transplant (KT) recipients selection.

From 13,245 potentially eligible KT recipients, we excluded those who met any of the following exclusion criteria: a history of a transplant received before 1997 (n = 1172); age at transplantation <18 years (n = 62); a follow-up < 30 days after KT (n = 489); or a diagnosis of cancer made during the 5 years preceding KT or within 30 days after KT (n = 104). Thus, the final population in study consisted of 11,418 subjects (Figure 1).

At each participating center, trained personnel collected appropriate information from clinical records and checked the quality of data for completeness and accuracy. Collected data included demographic and transplant characteristics of KT recipients, plus follow-up data. Cancer and vital status information was actively elicited from medical records till 31 December 2021. Since an active follow-up on return to dialysis, vital status, and cancer occurrence is planned on a yearly basis, there were some centers that had not yet updated the requested information as of December 2021; thus, for 3055 KT recipients (26.8%), the last information was collected in December 2020.

All tumor diagnoses (coded according to the International Classification of Diseases and Related Health Problems, 10th revision—ICD-10 [16]) were histologically confirmed. Multiple primary tumors were considered in site-specific analyses.

The ICD-10 codes used to define each cancer site or group are shown in Supplementary Table S1.

### Statistical Analysis

Person-years (PYs) at risk were accumulated from 30 days after KT, in 3 calendar periods: 1997 to 2004 (a); 2005 to 2012 (b); and 2013 to 2021 (c); roughly corresponding to: prevalent cyclosporine use (a); progressive switch to tacrolimus (b); and larger use of combinations with mTOR-inhibitors (c). In each period, PYs accruement was terminated at the date of cancer diagnosis, date of death, date of return to dialysis, date of loss to follow-up, or December 31 of the last year of the period, whichever occurred first. Overall, individuals were not censored at their first cancer diagnosis as they were still at risk of other types of cancers included. When considering site-specific analyses, after a cancer diagnosis patients did not further contribute follow-up time to the determination of PYs at risk for that specific tumor, but they continued to add follow-up time for other tumor sites/types.

To assess changes in cancer incidence rates (IRs) across periods, incidence rate ratios (IRRs) and corresponding 95% confidence intervals (CIs) were estimated using Poisson regression models adjusted for sex, age, residence area, and length of follow-up. For each model, P values for linear trends across periods were computed [14].

To compare cancer risk in KT recipients with that in the general population for each of the periods, standardized incidence ratios (SIRs) were calculated as the ratio of observed to expected numbers of cancer cases [17]. The expected numbers of cases were calculated by multiplying the PYs at risk among KT recipients by the corresponding population cancer IRs (as described elsewhere in more detail using data from Italian Cancer Registries) [5]. SIRs were standardized by sex, age (5-year groups), residence area, and calendar period. The 95% CIs for SIRs were calculated assuming a Poisson distribution [18]. We used Poisson regression to evaluate trends in SIRs, with the same adjustments used for the incidence models described above. All analyses were performed separately for all cancers combined and for each of the major cancer types. All tests were two-sided, with *p* < 0.05 considered statistically significant. All analyses were performed using SAS software (SAS Institute, Cary, NC, USA, v9.4).

## 3. Results

The 11,418 KT recipients (median age at KT 50 years; interquartile range, IQR: 39–58 years; 63.8% male) were followed for a total of 85,209 PYs of observation with a median follow-up time of 7.1 years (IQR: 3.9–10.6 years) (Table 1). Most KT recipients resided in northern Italy (56.7%), and the transplanted kidney was usually from a deceased donor (90.6%). Glomerulonephritis was the most common cause of kidney failure (36.9%), followed by polycystic kidney disease (17.0%) and pyelonephritis/interstitial nephritis (9.8%) (Table 1).

### 3.1. Internal Comparison

Table 2 presents IRs (per 1000 PYs) and unadjusted/adjusted IRRs for major cancer sites according to calendar periods of cancer diagnosis.

A total of 1646 post-transplant cancers were diagnosed, with IRs ranging from 15.5 per 1000 PYs in 1997–2004 to 21.0 per 1000 PYs in 2013–2021 (Table 2). The most common cancer type was nonmelanoma skin cancers (NMSC) (IR range 5.1-to-9.9 per 1000 PYs), followed by kidney cancer (IR range 0.9-to-1.6 per 1000 PYs), KS (IR range 0.4-to-2.5 per 1000 PYs), prostate cancer (IR range 0.7-to-1.4 per 1000 PYs), and lung cancer (IR range 0.7-to-1.5 per 1000 PYs). IRs ranged from 1.3 to 1.8 for all post-transplant lymphoproliferative diseases (PTLD) combined, with NHLs being the most common hematological malignancies (IR range 1.0-to-1.4 per 1000 PYs).

The unadjusted IRRs for all cancers combined (Table 2) showed a statistically significant increase in incidence rates over time as compared to the first study period, i.e., 1997–2004 (IRR = 1.29, 95% CI: 1.13–1.48 for 2005–2012; and IRR = 1.36, 95% CI: 1.18–1.57 for 2013–2021; P_trend_ < 0.01). When the analysis was restricted to the most common cancer types, an increased risk over time was noted for NMSC and for all solid tumors combined (including kidney and lung cancers), whereas a reduced incidence was noted for KS only.

Apart from KS, adjustment for sex, age, residence area, and length of follow-up elicited differing results. As compared to 1997–2004, IRRs for NMSC were significantly higher in both 2005–2012 (IRR = 1.36) and in 2013–2021 (IRR = 1.30), though the test for trend did not reach statistical significance. A significant reduction in incidence was noted for all cancers combined after excluding NMSC (IRR = 0.90 for 2005–2012 and IRR = 0.72 for 2013–2021; P_trend_ < 0.01). IRRs for KS maintained a significant downward trend over time (IRR = 0.37, 95% CI: 0.24–0.57 for 2005–2012 and IRR = 0.09, 95% CI: 0.04–0.18 for 2013–2021; P_trend_ < 0.01) (Table 2).

### 3.2. External Comparison

**Figure 2:**
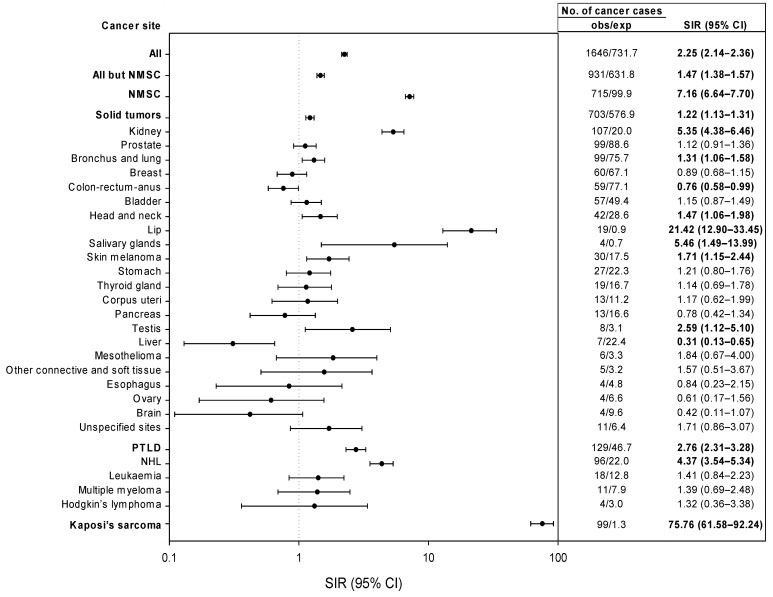
Standardized incidence ratios (SIR) and 95% confidence intervals (CI) for major cancer sites. Sites/types with <4 observed cases are not shown in figure. Abbreviations: NHL, non-Hodgkin lymphoma; NMSC, nonmelanoma skin cancer; obs/exp, observed/expected; PTLD, post-transplant lymphoproliferative diseases. Bold text indicates results that are statistically significant (*p* < 0.05).

SIRs of cancer risk in KT recipients as compared to the corresponding general population for the whole study period are illustrated in Figure 2.

For all cancers combined, a 2.3-fold higher risk (95% CI: 2.14–2.36) was documented. SIRs were particularly elevated for KS (SIR = 75.76, 95% CI: 61.58–92.24), NHL (SIR = 4.37, 95% CI: 3.54–5.34), NMSC (SIR = 7.16, 95% CI: 6.64–7.70), skin melanoma (SIR = 1.71, 95% CI: 1.15–2.44), kidney cancer (SIR = 5.35, 95% CI: 4.38–6.46), lung cancer (SIR = 1.31, 95% CI: 1.06–1.58), testis (SIR = 2.59, 95% CI: 1.12–5.10), and head and neck cancers (SIR = 1.47, 95% CI: 1.06–1.98), including lip (SIR = 21.42, 95% CI: 12.90–33.45) and salivary glands (SIR = 5.46, 95% CI: 1.49–13.99). Conversely, a significantly reduced risk was noted for cancers of the colon-rectum-anus (SIR = 0.76, 95% CI: 0.58–0.99) and liver (SIR = 0.31, 95% CI: 0.13–0.65).

In the analyses by period of cancer diagnosis, SIRs for all cancers combined were persistently elevated with decreasing magnitude over time, from 2.54 (95% CI: 2.26–2.85) in 1997–2004 to 1.99 (95% CI: 1.83–2.16) in 2013–2021 (P_trend_ < 0.01) (Table 3).

This trend was attenuated, but still statistically significant, after excluding NMSC (SIR = 1.96, 95% CI: 1.70–2.26 in 1997–2004 and SIR = 1.23, 95% CI: 1.10–1.38 in 2013–2021). Significant SIR declines emerged for KS (SIR = 189.16, 95% CI: 138.49–252.32 in 1997–2004 and SIR = 20.19, 95% CI: 9.68–37.14 in 2013–2021) and PTLD (SIR = 4.34, 95% CI: 3.01–6.07 in 1997–2004 and SIR = 2.37, 95% CI: 1.72–3.20 in 2013–2021), including NHL (SIR = 6.77, 95% CI: 4.42–9.92 in 1997–2004 and SIR = 3.74, 95% CI: 2.54–5.31 in 2013–2021). However, only the KS trend retained statistical significance after adjustment for sex, age, residence area, and length of follow-up (P_trend_ < 0.01).

## 4. Discussion

In this cohort study, we provided estimates of cancer incidence over a period of 25 years among Italian KT recipients. Although our analysis by periods showed—through an internal and external comparison—a decreasing trend in the incidence of all cancers combined among KT recipients, we found no significant changes in the incidence over time for the major cancer types, with the exception of Kaposi’s sarcoma.

The present study allowed us to update our previous estimates of cancer risk among Italian KT recipients [5], focusing on the analysis of time trends. Approximately 13% of KT recipients included in the analysis developed cancer during the considered follow-up period, equivalent to a 2.3-fold higher risk of developing cancer than the corresponding general population. We found very high excess risks for KS, NHL, lip cancer, and salivary gland cancer—in line with our previous studies [5] and investigations conducted in other high-income countries [1,2,3,12,13]—supporting an important role of viral infections in augmenting such risk in a context of drug-induced immunodeficiency [9]. Furthermore, the spectrum of virus-unrelated cancers for which we observed excess risks, such as skin melanoma and cancers of the lung and kidney, confirmed previous evidence of an increased risk presumably associated with unhealthy lifestyle behaviors (e.g., heavy alcohol consumption, tobacco smoking, or excessive sun exposure) and with factors related to the underlying cause of end-stage renal disease [12,13]. Noteworthy among malignancies frequently found in the general population, colorectal (SIR = 0.76) and liver (SIR = 0.31) cancers were not increased in KT recipients, but we noted a significantly reduced risk compared to the general population; a similar consideration could also be made for breast cancers, which showed a tendency to a decreased risk (SIR = 0.89), although not significant. Among the reasons for the lack of excess of these cancers in KT recipients, pre-transplant screening is likely to have played a prominent role.

We found a significant reduction over time in the incidence rates of all cancers combined excluding NMSC. Nevertheless, there was an increased incidence of NMSC, lung cancer, and kidney cancer in the unadjusted analysis. These trends disappeared after multivariate adjustment, and we did not also notice a trend in risks compared with the general population. Renal cell carcinoma (RCC) of native kidneys is a frequent malignancy among KT recipients [19]. The increased incidence over time of kidney cancer could partially reflect the changing prevalence of the risk factors affecting RCC (e.g., older age at KT, smoking, or excess body weight) [20]. Similarly, increasing age and changes in smoking habits contributed to the rising trend in lung cancer incidence. Although data on smoking were not available for this analysis, our findings highlighted that more emphasis should be placed in screening for smoking in the pre-transplant evaluation and during the follow-up visits after KT. A potential reason for the augmented rates of NMSC could be ascribed to the increased dermatological surveillance in more recent years among KT recipients [21,22]. However, our findings are in contrast with the reduced risk of cutaneous carcinomas (either squamous- or basal-cell carcinomas) in SOT recipients observed in recent decades by other studies conducted in the SOT setting [23,24].

We found a strong decline in KS incidence rates, clear also in the adjusted analysis. It is important to note that there might be some differences in the impact of oncogenic viruses on cancer development in patients of different countries. In this regard, the very high SIR for KS documented in the past decades was attributable to the high prevalence of infection with KS-associated herpes virus (KSHV) in various Mediterranean countries, including Italy [5,25,26]. Two nationwide investigations conducted in northern Europe (namely Norway and Sweden), where the prevalence of KSHV is extremely low, had too few cases to evaluate any longitudinal trends over time in KS incidence in the transplant population [23,27]. The decrease in KS risk that we observed over calendar time could reflect improvements in the management of KT recipients, presumably in the dosing and choice of immunosuppressive drugs, and possibly a role of pre-transplant screening [28,29]. Apart from KS, no significant changes over time in the incidence of other cancers emerged.

Long-term follow-up confirms that de novo cancers always occur, at any time, in a larger amount when compared to the general population, but their nature and distribution still seem uneven in different reports, depending on many different local factors [30]. There is a paucity of parallel findings showing changes in cancer risk over time in other investigations. A population-based study conducted in Ireland observed a decreasing trend in SIRs for overall cancer (excluding NMSC) after SOT between 1994 and 2014, although the test for trend was not statistically significant [31]. Furthermore, a recent study from the Finnish registry reported a clear decrease in cancer risk (SIR = 4.3 and 3.0 in 1987–1999 and 2000–2016, respectively) of 6548 SOT recipients [1]. However, the study design differed significantly from ours.

Although noteworthy differences in study design should be considered, our findings of no change in cancer incidence for all major cancer types are in line with those reported in a recent US investigation [14], and they highlight the need for improved interventions to limit the cancer burden in KT recipients which balance the need for cancer control with the maintenance of kidney graft function. Careful management of immunosuppression in the post-transplant setting remains crucial [32], and several drugs used as immunosuppressants may have direct effects on cancer development and progression [33]. For instance, a direct carcinogenic potential was documented for calcineurin inhibitors that are able to favor cancer cell invasiveness, dysregulating DNA repair mechanisms and the regulation of apoptosis, increasing the production of proangiogenic growth factor, and promoting transforming growth factor-beta functional expression [34]. In contrast, mammalian target of rapamycin inhibitors are able to interfere with cancer cell proliferation and new angiogenesis process, and their use has been associated with a lower incidence of malignancies such as KS or mantle cell lymphoma [35]. Nevertheless, in a context where KT recipients are generally treated with multidrug maintenance therapy, it is difficult to enucleate the single effect of a particular drug from the effect of the overall immunosuppressive regimen and to assess cancer risks in relation to different immunosuppressive treatments. In our study, the immunosuppression regimes varied across the participating centers, and any change in the policy of standard immunosuppressive protocols to be used (e.g., the introduction of new drugs, switching to an alternative drug regimen) could have occurred at different times in the centers considered, and often, the therapeutic approach in specific subgroups of patients may have changed over time. Therefore, we could not stratify the analysis using clear fixed-year milestones to investigate the role of immunosuppression on cancer incidence trends over time.

Although the increased risk of cancer after KT has been well described and characterized by observational and registry data, few investigations have explored recent trends in cancer incidence among individuals who received SOT. To the best of our knowledge, our study is the largest cohort to provide estimates of recent variations in the occurrence of post-transplant cancers among KT recipients in a southern European population. The strengths of our study are the large sample size that allowed for analyses by cancer type/site, the availability of data over 25 years, and the multicenter nature of the cohort.

### 4.1. Limitations

We could not explore which individual factors may have contributed to the observed temporal trends, since information about patients’ comorbidities—which are highly prevalent in this population—as well as data about cancer screening and some lifestyle habits (e.g., smoking, alcohol abuse), are not regularly collected in Italian KT centers with the same details and completeness and were not included in the shared form used in this multicenter study to ensure uniform data collection. Moreover, when these individual data are eventually available, their use is restricted by data protection regulations. The lack of such information, as well as of several clinical data, represents a substantial drawback. Furthermore, since all cancer diagnoses were registered on the basis of medical records, a partial lack of completeness of cancer case ascertainment could be possible. However, rigorous clinical follow-up of these patients (at least one visit per year) is likely to minimize the risk of cancer underreporting, ensuring that it is definitely not higher than in the general population.

Due to the dynamic nature of the cohort, the number of members could vary over time and the duration of follow-up varied between individuals, which may have led to survival bias. However, all models were adjusted for the length of follow-up in order to overcome, at least in part, this limitation.

Finally, despite the large sample size of patients and long follow-up, for specific cancers, the small numbers were a drawback for period analysis.

### 4.2. Future Directions

The findings of this study highlight the need for further research to identify valuable clues about the role of the immune system in cancer etiology while helping clinicians and public health advisers to develop early detection and screening approaches for these patients. Moreover, further investigations are warranted to evaluate site-specific cancer mortality in transplant recipients with long-term follow-up, and, whether progress in cancer mortality in the general population is mirrored in the transplant recipients population.

## 5. Conclusions

Our findings revealed a significant reduction in the risk of KS over time among Italian KT recipients. Apart from KS, there were no changes over time in the incidence of other cancers. It remains unclear to what extent immunosuppression has influenced cancer incidence trends.

As the transplant population swells, high cancer risk among recipients represents an increasing public health concern. The knowledge of modifiable risk factors and the availability of individualized screening may improve prevention and, therefore, the clinical outcome of KT recipients [36]. Close advice in cancer screening and prevention has long been suggested, but recently more tailored and intensified prognostic and predictive analyses have been suggested to better guide treatment modulation in high-risk patients [37,38].

## Figures and Tables

**Table 1:** Characteristics of 11,418 patients who underwent kidney transplantation.

	All Patients	
	N	%
**Sex**		
Male	7286	63.8
Female	4132	36.2
**Age at transplant (years)**		
18–39	2907	25.5
40–49	2682	23.5
50–59	3439	30.1
≥60	2390	20.9
Median (IQR)	50 (39–58)	
**Calendar year at transplant**		
1997–2004	5737	50.3
2005–2012	3893	34.1
2013–2021	1788	15.7
**Area of residence**		
Northern Italy	6471	56.7
Central Italy	1405	12.3
Southern Italy	3489	30.5
Abroad	53	0.5
**Status of the donor**		
Alive	1079	9.4
Deceased	10,339	90.6
**Primary cause of kidney failure**		
Glomerulonephritis	4212	36.9
Polycystic kidney disease	1936	17.0
Pyelonephritis/Interstitial nephritis	1116	9.8
Hypertensive nephropathy/vascular disease	691	6.0
Diabetes	604	5.3
Congenital	394	3.4
Uncertain	1781	15.6
Other	684	6.0
**Follow-up (years)**		
Median (IQR)	7.1 (3.9–10.6)	
Total person-years	85,208.7	

Abbreviations: IQR, interquartile range.

**Table 2:** Incidence rates (IR) per 1000 person-years and incidence rate ratios (IRR) for major cancer sites, by calendar period of cancer diagnosis.

	1997–2004		2005–2012		2013–2021		2005–2012	2013–2021		2005–2012	2013–2021	
Cancer Site	No.	IR (SE)	No.	IR (SE)	No.	IR (SE)	Unadjusted IRR (95% CI) ^a^		P_trend_	Adjusted IRR (95% CI) ^a,b^		P_trend_
All	289	15.5 (0.9)	771	19.9 (0.7)	586	21.0 (0.9)	**1.29 (1.13–1.48)**	**1.36 (1.18–1.57)**	**<0.01**	1.05 (0.92-1.21)	0.92 (0.80-1.06)	0.11
All but NMSC	194	10.4 (0.7)	427	11.0 (0.5)	310	11.1 (0.6)	1.06 (0.90–1.26)	1.07 (0.90–1.28)	0.48	0.90 (0.76–1.07)	**0.72 (0.60–0.87)**	**<0.01**
NMSC	95	5.1 (0.5)	344	8.9 (0.5)	276	9.9 (0.6)	**1.75 (1.40–2.20)**	**1.95 (1.54–2.46)**	**<0.01**	**1.36 (1.08–1.71)**	**1.30 (1.02–1.65)**	0.11
Solid tumors	114	6.1 (0.6)	332	8.6 (0.5)	257	9.2 (0.6)	**1.41 (1.14–1.74)**	**1.51 (1.21–1.88)**	**<0.01**	1.17 (0.95–1.46)	1.00 (0.80–1.26)	0.62
Kidney	17	0.9 (0.2)	46	1.2 (0.2)	44	1.6 (0.2)	1.31 (0.75–2.28)	1.74 (0.99–3.04)	**0.04**	1.30 (0.74–2.29)	1.50 (0.84–2.67)	0.17
Prostate	13	0.7 (0.2)	53	1.4 (0.2)	33	1.2 (0.2)	**1.98 (1.08–3.63)**	1.71 (0.90–3.26)	0.19	1.45 (0.78–2.67)	0.97 (0.50–1.86)	0.49
Bronchus and lung	14	0.7 (0.2)	44	1.1 (0.2)	41	1.5 (0.2)	1.52 (0.83–2.78)	**1.97 (1.07–3.61)**	**0.03**	1.14 (0.62–2.09)	1.01 (0.55–1.89)	0.88
Breast	9	0.5 (0.2)	33	0.9 (0.1)	18	0.6 (0.2)	1.78 (0.85–3.71)	1.34 (0.60–2.99)	0.65	1.62 (0.76–3.42)	1.06 (0.46–2.41)	0.81
Colon-rectum-anus	7	0.4 (0.1)	29	0.8 (0.1)	23	0.8 (0.2)	2.00 (0.88–4.57)	2.21 (0.95–5.15)	0.09	1.54 (0.67–3.54)	1.39 (0.59–3.29)	0.63
Bladder	12	0.6 (0.2)	30	0.8 (0.1)	15	0.5 (0.1)	1.21 (0.62–2.36)	0.84 (0.39–1.80)	0.58	0.97 (0.49–1.92)	0.63 (0.29–1.37)	0.20
Head and neck	6	0.3 (0.1)	19	0.5 (0.1)	17	0.6 (0.1)	1.53 (0.61–3.83)	1.91 (0.75–4.84)	0.17	1.29 (0.51–3.26)	1.32 (0.51–3.43)	0.62
Skin melanoma	7	0.4 (0.1)	13	0.3 (0.1)	10	0.4 (0.1)	0.90 (0.36–2.25)	0.96 (0.37–2.52)	0.96	0.92 (0.36–2.34)	0.99 (0.36–2.70)	0.99
Stomach	5	0.3 (0.1)	14	0.4 (0.1)	8	0.3 (0.1)	1.35 (0.49–3.76)	1.07 (0.35–3.28)	0.98	1.01 (0.36–2.84)	0.49 (0.16–1.52)	0.13
PTLD	34	1.8 (0.3)	52	1.3 (0.2)	43	1.5 (0.2)	0.74 (0.48–1.14)	0.85 (0.54–1.33)	0.56	0.69 (0.44–1.07)	0.71 (0.44–1.13)	0.19
NHL	26	1.4 (0.3)	39	1.0 (0.2)	31	1.1 (0.2)	0.73 (0.44–1.19)	0.80 (0.48–1.35)	0.47	0.69 (0.41–1.14)	0.68 (0.39–1.17)	0.19
Kaposi’s sarcoma	46	2.5 (0.4)	43	1.1 (0.2)	10	0.4 (0.1)	**0.45 (0.30–0.69)**	**0.15 (0.07–0.29)**	**<0.01**	**0.37 (0.24–0.57)**	**0.09 (0.04–0.18)**	**<0.01**

^a^ Reference group is the period 1997–2004; ^b^ Adjusted for sex, age, area of residence, and length of follow-up; Abbreviations: CI, confidence intervals; NHL, non-Hodgkin lymphoma; NMSC, nonmelanoma skin cancer; PTLD, post-transplant lymphoproliferative diseases; SE, standard error. Bold text indicates statistically significant (*p* < 0.05) results.

**Table 3:** Standardized incidence ratios (SIR) and 95% confidence intervals (CI) for major cancer sites by calendar period of cancer diagnosis.

	1997–2004	2005–2012	2013–2021	P_trend_	
Cancer Site	SIR (95% CI)	SIR (95% CI)	SIR (95% CI)	Unadjusted	Adjusted ^a^
All	**2.54 (2.26–2.85)**	**2.38 (2.21–2.55)**	**1.99 (1.83–2.16)**	**<0.01**	**<0.01**
All but NMSC	**1.96 (1.70–2.26)**	**1.52 (1.38–1.67)**	**1.23 (1.10–1.38)**	**<0.01**	**<0.01**
NMSC	**6.40 (5.18–7.83)**	**8.05 (7.23–8.95)**	**6.52 (5.77–7.33)**	0.40	0.42
Solid tumors	**1.27 (1.04–1.52)**	**1.29 (1.16–1.44)**	1.12 (0.99–1.26)	0.14	0.17
Kidney	**5.33 (3.10–8.53)**	**5.17 (3.78–6.89)**	**5.56 (4.04–7.46)**	0.81	0.38
Prostate	1.28 (0.68–2.19)	1.34 (1.00–1.75)	0.85 (0.59–1.20)	0.07	0.09
Bronchus and lung	1.16 (0.64–1.95)	1.33 (0.96–1.78)	1.35 (0.97–1.83)	0.68	0.86
Breast	0.73 (0.33–1.38)	1.08 (0.74–1.52)	0.74 (0.44–1.17)	0.78	0.71
Colon-rectum-anus	0.61 (0.24–1.25)	0.85 (0.57–1.22)	0.73 (0.47–1.10)	0.87	0.83
Bladder	1.66 (0.86–2.90)	1.38 (0.93–1.97)	0.73 (0.41–1.21)	**0.02**	0.12
Head and neck	1.08 (0.40–2.36)	1.49 (0.90–2.33)	1.64 (0.96–2.63)	0.40	0.45
Skin melanoma	2.42 (0.97–5.00)	1.66 (0.89–2.84)	1.47 (0.70–2.70)	0.34	0.58
Stomach	1.34 (0.44–3.13)	1.44 (0.79–2.42)	0.90 (0.39–1.77)	0.38	0.15
PTLD	**4.34 (3.01–6.07)**	**2.51 (1.87–3.29)**	**2.37 (1.72–3.20)**	**0.02**	0.11
NHL	**6.77 (4.42–9.92)**	**3.96 (2.82–5.42)**	**3.74 (2.54–5.31)**	**0.04**	0.17
Kaposi’s sarcoma	**189.16 (138.49–252.32)**	**75.66 (54.76–101.92)**	**20.19 (9.68–37.14)**	**<0.01**	**<0.01**

^a^ Adjusted for sex, age, area of residence, and length of follow-up. Abbreviations: NHL, non-Hodgkin lymphoma; NMSC, nonmelanoma skin cancer; PTLD, post-transplant lymphoproliferative diseases. Bold text indicates statistically significant (*p* < 0.05) results.

## Data Availability

The data or code that support the findings of this study are available from the corresponding author upon reasonable request.

## Supplementary Materials

The following supporting information can be downloaded at: https://www.mdpi.com/article/10.3390/cancers15041347/s1, Table S1: Cancer site and group definitions by the International Classification of Diseases and Related Health Problems, 10th revision (ICD-10).

## Appendix A

Others of the Italian Transplant & Cancer Cohort Study: Lucia Fratino, Federica Toffolutti (Centro di Riferimento Oncologico di Aviano IRCCS, Aviano, Italy); Alessandro Agresta, Enrico Girardi, Alessandra Nappo (INMI “L. Spallanzani” IRCCS, Rome, Italy); Elisa Boccalon, Caterina di Bella, Erica Nuzzolese, Paolo Rigotti (Padova University Hospital, Padova, Italy); Antonio Lavacca (Turin University Hospital, Turin, Italy); Valeriana Colombo, Marialuisa Querques (S.C. Nefrologia, Grande Ospedale Metropolitano Niguarda, Milano, Italy); Stefano Possenti, Silvio Sandrini (Spedali Civili of Brescia, Brescia, Italy); Laura Panicali (Divisione di Nefrologia Dialisi ed Ipertensione, S. Orsola-Malpighi Hospital, Bologna, Italy); Chiara Valentini (Ospedale S. Maria delle Croci, Ravenna, Italy); Elena Cremaschi, Umberto Maggiore, Alessandra Palmisano, Carlo Pellegrino, Carmelo Puliatti (Parma University Hospital, Parma, Italy); Giuseppe Grandaliano, Maria Paola Salerno, Elisabetta Schifano, Patrizia Silvestri, Gionata Spagnoletti (Policlinico “A. Gemelli”, Rome, Italy); Carlo Maria Alfieri, Giuseppe Castellano, Evaldo Favi, Mariano Ferraresso, Samuele Iesari (Fondazione IRCCS Ca’ Granda Ospedale Maggiore Policlinico, Milano, Italy); Daniela Corona, Costanza Distefano, Pierfrancesco Veroux (Policlinico of Catania, Catania, Italy); Francesca Blasi, Francesca Romano, Luca Toti (Tor Vergata University, Rome, Italy); Cristina Cornella, Guido Merlotti, Marco Quaglia (University of Piemonte Orientale, “Maggiore della Carità” University Hospital, Novara, Italy); Maria Cristina Maresca (Treviso Hospital, Treviso, Italy); Loreto Gesualdo (Renal, Dialysis and Transplant Unit, Department of Emergency and Organ Transplant, University of Bari, Polyclinic, Bari, Italy); Elisabetta Carta, Gian Benedetto Piredda (Azienda Ospedaliera Brotzu, Cagliari, Italy); Marco De Cicco, Giuseppe Ietto, Domenico Iovino, Francesco Perna (Azienda Ospedaliera Ospedale di Circolo e Fondazione Macchi, Varese, Italy); Laura Lancione, Alessandra Panarese (University of L’Aquila, L’Aquila, Italy); Antonio Amato, Barbara Buscemi (Civico and Di Cristina Hospital, Palermo, Italy).

## References

[1] Friman T.K., Jäämaa-Holmberg S., Åberg F., Helanterä I., Halme M., Pentikäinen M.O., Nordin A., Lemström K.B., Jahnukainen T., Räty R. (2022). Cancer risk and mortality after solid organ transplantation: A population-based 30-year cohort study in Finland. Int. J. Cancer.

[2] Acuna S.A. (2018). Etiology of increased cancer incidence after solid organ transplantation. Transplant. Rev..

[3] Engels E.A., Pfeiffer R.M., Fraumeni J.F., Kasiske B.L., Israni A.K., Snyder J.J., Wolfe R.A., Goodrich N.P., Bayakly A.R., Clarke C.A. (2011). Spectrum of Cancer Risk Among US Solid Organ Transplant Recipients. JAMA.

[4] Au E., Wong G., Chapman J.R. (2018). Cancer in kidney transplant recipients. Nat. Rev. Nephrol..

[5] Piselli P., Serraino D., Segoloni G.P., Sandrini S., Piredda G.B., Scolari M.P., Rigotti P., Busnach G., Messa P., Donati D. (2013). Risk of de novo cancers after transplantation: Results from a cohort of 7217 kidney transplant recipients, Italy 1997–2009. Eur. J. Cancer.

[6] Miao Y., Everly J.J., Gross T.G., Tevar A.D., First M.R., Alloway R.R., Woodle E.S. (2009). De Novo Cancers Arising in Organ Transplant Recipients are Associated With Adverse Outcomes Compared With the General Population. Transplantation.

[7] Au E.H., Chapman J.R., Craig J.C., Lim W.H., Teixeira-Pinto A., Ullah S., McDonald S., Wong G. (2019). Overall and Site-Specific Cancer Mortality in Patients on Dialysis and after Kidney Transplant. J. Am. Soc. Nephrol..

[8] Taborelli M., Serraino D., Cimaglia C., Furian L., Biancone L., Busnach G., Todeschini P., Bossini N., Iaria M., Campise M.R. (2021). The impact of cancer on the risk of death with a functioning graft of Italian kidney transplant recipients. Am. J. Transplant..

[9] Schulz T.F. (2009). Cancer and viral infections in immunocompromised individuals. Int. J. Cancer.

[10] Piselli P., Verdirosi D., Cimaglia C., Busnach G., Fratino L., Ettorre G.M., De Paoli P., Citterio F., Serraino D. (2014). Epidemiology of de novo malignancies after solid-organ transplantation: Immunosuppression, infection and other risk factors. Best Pract. Res. Clin. Obstet. Gynaecol..

[11] Engels E.A. (2017). Cancer in Solid Organ Transplant Recipients: There Is Still Much to Learn and Do. Am. J. Transplant..

[12] Chapman J.R., Webster A., Wong G. (2013). Cancer in the Transplant Recipient. Cold Spring Harb. Perspect. Med..

[13] Cheung C.Y., Tang S.C.W. (2018). An update on cancer after kidney transplantation. Nephrol. Dial. Transplant..

[14] Blosser C.D., Haber G., Engels E.A. (2021). Changes in cancer incidence and outcomes among kidney transplant recipients in the United States over a thirty-year period. Kidney Int..

[15] Nordin A., Åberg F., Pukkala E., Pedersen C.R., Storm H.H., Rasmussen A., Bennet W., Olausson M., Wilczek H., Ericzon B.-G. (2017). Decreasing incidence of cancer after liver transplantation—A Nordic population-based study over 3 decades. Am. J. Transplant..

[16] WHO (1992). International Classification of Diseases and Related Health Problems (10th Revision, ICD-10-CM).

[17] Breslow N.E., Day N.E. (1987). Statistical Methods in Cancer Research, Volume II. The Design and Analysis of Cohort Studies.

[18] Ulm K. (1990). A simple method to calculate the confidence interval of a standardized mortality ratio (SMR). Am. J. Epidemiol..

[19] Dahle D.O., Skauby M., Langberg C.W., Brabrand K., Wessel N., Midtvedt K. (2021). Renal Cell Carcinoma and Kidney Transplantation: A Narrative Review. Transplantation.

[20] Karami S., Yanik E.L., Moore L.E., Pfeiffer R.M., Copeland G., Gonsalves L., Hernandez B.Y., Lynch C.F., Pawlish K., Engels E.A. (2016). Risk of Renal Cell Carcinoma Among Kidney Transplant Recipients in the United States. Am. J. Transplant..

[21] Welch H.G., Mazer B.L., Adamson A.S. (2021). The Rapid Rise in Cutaneous Melanoma Diagnoses. N. Engl. J. Med..

[22] Acuna S.A., Lam W., Daly C., Kim S.J., Baxter N.N. (2018). Cancer evaluation in the assessment of solid organ transplant candidates: A systematic review of clinical practice guidelines. Transplant. Rev..

[23] Rizvi S.M.H., Aagnes B., Holdaas H., Gude E., Boberg K.M., Bjørtuft Ø., Helsing P., Leivestad T., Møller B., Gjersvik P. (2017). Long-term Change in the Risk of Skin Cancer After Organ Transplantation: A Population-Based Nationwide Cohort Study. JAMA Dermatol..

[24] Menzies S., O’Leary E., Callaghan G., Galligan M., Deady S., Gadallah B., Lenane P., Lally A., Houlihan D., Morris P. (2019). Declining incidence of keratinocyte carcinoma in organ transplant recipients. Br. J. Dermatol..

[25] García-Astudillo L.A., Leyva-Cobián F. (2006). Human herpesvirus-8 infection and Kaposi’s sarcoma after liver and kidney transplantation in different geographical areas of Spain. Transpl. Immunol..

[26] Serraino D., Piselli P., Angeletti C., Minetti E., Pozzetto A., Civati G., Bellelli S., Farchi F., Citterio F., Rezza G. (2005). Risk of Kaposi’s sarcoma and of other cancers in Italian renal transplant patients. Br. J. Cancer.

[27] Krynitz B., Edgren G., Lindelöf B., Baecklund E., Brattström C., Wilczek H., Smedby K.E. (2013). Risk of skin cancer and other malignancies in kidney, liver, heart and lung transplant recipients 1970 to 2008—A Swedish population-based study. Int. J. Cancer.

[28] Cahoon E.K., Linet M.S., Clarke C.A., Pawlish K.S., Engels E.A., Pfeiffer R.M. (2018). Risk of Kaposi sarcoma after solid organ transplantation in the United States. Int. J. Cancer.

[29] Piselli P., Taborelli M., Cimaglia C., Serraino D., Italian Transplant & Cancer Cohort Study (2019). Decreased incidence of Kaposi sarcoma after kidney transplant in Italy and role of mTOR-inhibitors: 1997–2016: Risk of Kaposi sarcoma after solid organ transplantation in the United States. Int. J. Cancer.

[30] Fröhlich F.A., Halleck F., Lehner L., Schrezenmeier E.V., Naik M., Schmidt D., Khadzhynov D., Kast K., Budde K., Staeck O. (2020). De-novo malignancies after kidney transplantation: A long-term observational study. PLoS ONE.

[31] O’Neill J.P., Sexton D.J., O’Leary E., O’Kelly P., Murray S., Deady S., Daly F., Williams Y., Dean B., Fitzgerald C. (2019). Post-transplant malignancy in solid organ transplant recipients in Ireland, The Irish Transplant Cancer Group. Clin. Transplant..

[32] Wojciechowski D., Wiseman A. (2021). Long-Term Immunosuppression Management: Opportunities and Uncertainties. Clin. J. Am. Soc. Nephrol..

[33] Krisl J.C., Doan V.P. (2017). Chemotherapy and Transplantation: The Role of Immunosuppression in Malignancy and a Review of Antineoplastic Agents in Solid Organ Transplant Recipients. Am. J. Transplant..

[34] Cangemi M., Montico B., Faè D.A., Steffan A., Dolcetti R. (2019). Dissecting the Multiplicity of Immune Effects of Immunosuppressive Drugs to Better Predict the Risk of de novo Malignancies in Solid Organ Transplant Patients. Front. Oncol..

[35] De Fijter J.W. (2017). Cancer and mTOR Inhibitors in Transplant Recipients. Transplantation.

[36] Al-Adra D., Al-Qaoud T., Fowler K., Wong G. (2022). De Novo Malignancies after Kidney Transplantation. Clin. J. Am. Soc. Nephrol..

[37] Huang B., Huang M., Zhang C., Yu Z., Hou Y., Miao Y., Chen Z. (2022). Individual dynamic prediction and prognostic analysis for long-term allograft survival after kidney transplantation. BMC Nephrol..

[38] Turshudzhyan A. (2021). Post-renal transplant malignancies: Opportunities for prevention and early screening. Cancer Treat. Res. Commun..

