# Supplementary material for: Variation in Post-Transplant Cancer Incidence among Italian Kidney Transplant Recipients over a 25-Year Period
Source: Cancers (Basel). 2023 Feb 20;15(4):1347. doi: 10.3390/cancers15041347 (PMC9954633; doi:10.3390/cancers15041347)
Supplement: Supplementary file 1 [file cancers-15-01347-s001.zip › cancers-2149072-supplementary.pdf]

Supplementary Table S1. Cancer site and group definitions by the International Classification of Diseases and Related Health Problems, 10th revision (ICD-10).

Site/group name	ICD-10 code
All	C00-C97, D09.0, D30.3, D41.4
All but NMSC	C00-C97 (excl. C44), D09.0, D30.3, D41.4
Solid tumors	C00-C80 (excl. C44, C46)
Head and neck	C00-14, C30-32
Lip	C00
Salivary glands	C07-08
Esophagus	C15
Stomach	C16
Colon-rectum-anus	C18-21
Liver	C22
Pancreas	C25
Bronchus and lung	C34
Skin melanoma	C43
NMSC	C44
Mesothelioma	C45
Kaposi's sarcoma	C46
Other connective and soft tissue	C49
Breast	C50
Corpus uteri	C54-55
Ovary	C56
Prostate	C61
Testis	C62
Kidney	C64
Bladder	C67, D09.0, D30.3, D41.4
Brain	C71
Thyroid gland	C73
Unspecified sites	C76-80
PTLD	C81-96
Hodgkin lymphoma	C81
Non-Hodgkin lymphoma	C82-85, C96
Multiple myeloma	C90
Leukaemia	C91-95

Abbreviations: NMSC, nonmelanoma skin cancer; PTLD, posttransplant lymphoproliferative diseases.
